# Supplementary material for: An open-source deep learning network AVA-Net for arterial-venous area segmentation in optical coherence tomography angiography
Source: Commun Med (Lond). 2023 Apr 17;3:54. doi: 10.1038/s43856-023-00287-9 (PMC10110614; doi:10.1038/s43856-023-00287-9)
Supplement: Supplementary file 1 — Description of Additional Supplementary Files [file 43856_2023_287_MOESM1_ESM.pdf]

Description of Additional Supplementary Files

File Name: Supplementary Data 1

Description: Numerical results of individual subjects underlying the graphs and charts presented in the main Fig 5 and Fig 6
