# Supplementary material for: An open-source deep learning network AVA-Net for arterial-venous area segmentation in optical coherence tomography angiography
Source: Commun Med (Lond). 2023 Apr 17;3:54. doi: 10.1038/s43856-023-00287-9 (PMC10110614; doi:10.1038/s43856-023-00287-9)
Supplement: Supplementary file 4 — Reporting Summary [file 43856_2023_287_MOESM4_ESM.pdf]

nature portfolio

Corresponding author(s): Professor Xincheng Yao (University of Illinois at Chicago)

Last updated by author(s): Mar 22, 2023

Reporting Summary

Nature Portfolio wishes to improve the reproducibility of the work that we publish. This form provides structure for consistency and transparency in reporting. For further information on Nature Portfolio policies, see our Editorial Policies and the Editorial Policy Checklist.

Statistics

For all statistical analyses, confirm that the following items are present in the figure legend, table legend, main text, or Methods section.

n/a	Confirmed	
☐	☒	The exact sample size (*n*) for each experimental group/condition, given as a discrete number and unit of measurement
☐	☒	A statement on whether measurements were taken from distinct samples or whether the same sample was measured repeatedly
☐	☒	The statistical test(s) used AND whether they are one- or two-sided<br>*Only common tests should be described solely by name; describe more complex techniques in the Methods section.*
☐	☒	A description of all covariates tested
☐	☒	A description of any assumptions or corrections, such as tests of normality and adjustment for multiple comparisons
☐	☒	A full description of the statistical parameters including central tendency (e.g. means) or other basic estimates (e.g. regression coefficient) AND variation (e.g. standard deviation) or associated estimates of uncertainty (e.g. confidence intervals)
☐	☒	For null hypothesis testing, the test statistic (e.g. *F*, *t*, *r*) with confidence intervals, effect sizes, degrees of freedom and *P* value noted<br>*Give P values as exact values whenever suitable.*
☒	☐	For Bayesian analysis, information on the choice of priors and Markov chain Monte Carlo settings
☒	☐	For hierarchical and complex designs, identification of the appropriate level for tests and full reporting of outcomes
☒	☐	Estimates of effect sizes (e.g. Cohen's *d*, Pearson's *r*), indicating how they were calculated

Our web collection on statistics for biologists contains articles on many of the points above.

Software and code

Policy information about availability of computer code

Data collection: ReVue software interface (Optovue) for AngioVue SD-OCT device (Optovue, Fremont, CA, USA)

Data analysis: Python (v3.9.6) using Keras (2.6.0) with Tensorflow (v2.6.0) backend, MATLAB R2021b

For manuscripts utilizing custom algorithms or software that are central to the research but not yet described in published literature, software must be made available to editors and reviewers. We strongly encourage code deposition in a community repository (e.g. GitHub). See the Nature Portfolio guidelines for submitting code & software for further information.

Data

Policy information about availability of data

All manuscripts must include a data availability statement. This statement should provide the following information, where applicable:

- Accession codes, unique identifiers, or web links for publicly available datasets
- A description of any restrictions on data availability
- For clinical datasets or third party data, please ensure that the statement adheres to our policy

The source data, i.e., the numerical results underlying the graphs and charts presented in the main figures, is provided as Supplementary Data 1. Training and test dataset images may be obtained from the corresponding author upon reasonable request.

Human research participants

Policy information about studies involving human research participants and Sex and Gender in Research.

Reporting on sex and gender	Sex of the subjects were considered in this study. We used χ2 tests to assess the distribution of sex among different groups.
Population characteristics	Subjects who were 18 years of age or older met the inclusion criteria. In addition, diabetic patients having a diagnosis of Type II diabetes mellitus met the inclusion criteria for our diabetic cohort. Subjects with macular edema, proliferative DR, previous vitrectomy surgery, history of other ocular disorders other than cataracts or minor refractive error, and ungradable and low-quality OCT pictures were exclusion criterions.
Recruitment	Control subjects and diabetic patients without and with DR in different stages were recruited from the UIC retina clinic. The patients present in this study are representative of a university population of diabetic patients who require clinical diagnosis and management of DR.
Ethics oversight	The study was approved by the Institutional Review Board (IRB) of the University of Illinois at Chicago (UIC) and is in adherence to the ethical standards set forth in the Declaration of Helsinki.

Note that full information on the approval of the study protocol must also be provided in the manuscript.

Field-specific reporting

Please select the one below that is the best fit for your research. If you are not sure, read the appropriate sections before making your selection.

☒ Life sciences ☐ Behavioural & social sciences ☐ Ecological, evolutionary & environmental sciences

For a reference copy of the document with all sections, see nature.com/documents/nr-reporting-summary-flat.pdf

Life sciences study design

All studies must disclose on these points even when the disclosure is negative.

Sample size	The sample size for similar studies in the literature is usually greater than 10.
Data exclusions	Subjects with macular edema, nonproliferative DR (NPDR) levels higher than mild NPDR, previous vitrectomy surgery, history of other ocular disorders other than cataracts or minor refractive error, and ungradable and low-quality OCT pictures were exclusion criterions.
Replication	Since our study is about image processing, it does not apply.
Randomization	It is not relevant to our study because a board-certified retina specialist classified the patients as NoDR or mild NPDR according to the Early Treatment Diabetic Retinopathy Study (ETDRS) staging system.
Blinding	It is not relevant to our study because a board-certified retina specialist classified the patients as NoDR or mild NPDR according to the Early Treatment Diabetic Retinopathy Study (ETDRS) staging system.

Reporting for specific materials, systems and methods

We require information from authors about some types of materials, experimental systems and methods used in many studies. Here, indicate whether each material, system or method listed is relevant to your study. If you are not sure if a list item applies to your research, read the appropriate section before selecting a response.

Materials & experimental systems

n/a	Involved in the study	
☒	☐	Antibodies
☒	☐	Eukaryotic cell lines
☒	☐	Palaeontology and archaeology
☒	☐	Animals and other organisms
☒	☐	Clinical data
☒	☐	Dual use research of concern

Methods

n/a	Involved in the study	
☒	☐	ChIP-seq
☒	☐	Flow cytometry
☒	☐	MRI-based neuroimaging
